# Effectiveness of ginkgo diterpene lactone meglumine on cognitive function in patients with acute ischemic stroke

**DOI:** 10.1515/med-2024-0908

**Published:** 2024-03-13

**Authors:** Meini Zhang, Xiao Hu, Tao Wang, Xianghong Liu

**Affiliations:** General Practice, Xi’an Medical University, Xi’an, 710000, China; Department of Pediatrics, Yan’an University Affiliated Hospital, Yan’an, 716000, China; Department of Internal Neurology, Shaanxi Provincial People’s Hospital, Xi’an, 710068, China; Department of Neurological Rehabilitation, Xi’an Gaoxin Hospital, Rongshang 10th District, 74 Zhuque Street South Section, Yanta District, Xi’an City, Shaanxi Province, 710000, China

**Keywords:** acute ischemic stroke, ginkgo diterpene lactone meglumine, PSCI, nerve function, cognitive function

## Abstract

**Objective:**

To explore the efficacy of ginkgo diterpene lactone (GDLM) on cognitive function in patients with acute ischemic stroke (AIS).

**Methods:**

A total of 126 patients with AIS in Shaanxi Provincial People’s Hospital from July 2019 to December 2020 were collected and randomly divided into the control group and treatment group (*n* = 63). All patients received conventional treatment, on which 25 mg/day GDLM was administered in the treatment group. Coagulation and inflammation indexes, National Institutes of Health Stroke Scale (NIHSS) and activities of daily living scale (ADL) scores were measured before and 14 days after treatment. NIHSS and ADL scores were performed again after 3 months. Cognitive function was assessed by Montréal Cognitive Assessment (MoCA) score, Mini-Mental State Examination (MMSE) score, and potential P300.

**Results:**

After 14 days of treatment, all biochemical indices were lower than before treatment (*P* < 0.05). The NIHSS and ADL scores of the treatment group were significantly better than those of the control group after treatment (*P* < 0.05). The MoCA and MMSE scores of the treatment group improved more significantly compared with the control group (*P* < 0.05). After treatment, the P300 indexes of both groups were significantly better than before treatment (*P* < 0.05).

**Conclusion:**

Conventional treatment of AIS combined with GDLM can effectively improve the cognitive function of patients, which is worthy of clinical recommendation.

## 1 Introduction

Stroke has emerged as a significant global public health issue as the population is aging dramatically more quickly. Stroke is the major cause of mortality and disability among Chinese citizens, and China has the highest rate of stroke patients worldwide. According to the data of Global Burden of Disease Study, the incidence of stroke in China has decreased from 222/100,000 in 2005 to 201/100,000 in 2019. However, the prevalence of stroke is still on the rise [1]. Stroke not only affects neurological functions such as motor, sensory, and swallowing, but also impairs cognitive functions including executive functions, memory, and learning. Post-stroke cognitive impairment (PSCI), specifically refers to a group of syndromes of varying types and degrees of cognitive impairment that meet the diagnostic criteria for cognitive impairment within 6 months of stroke. Numerous studies have shown that approximately one-third of stroke patients experience varying degrees of cognitive impairment [2,3]. Because it severely impacts patients’ independence, social involvement, quality of life, and long-term prognosis, stroke is a focus of international study and clinical intervention. It is also a substantial contributor to the contemporary burden of stroke.

Folium Ginkgo is the leaf of *Ginkgo biloba*, which is widely used as a traditional Chinese medicine for the treatment of cardiovascular ischemic diseases. Several studies in recent years have reported its efficacy in the treatment of various diseases such as cognitive impairment, glaucoma, and acute cerebral hemorrhage [4–6]. Ginkgo diterpene lactone meglumine (GDLM) are organic compounds made from *G. biloba* and contain ginkgolide A, B, and K as active components. They are effective inhibitors of platelet aggregation, suppressors of inflammatory responses, and preventers of cell membrane damage because they are natural antagonists of the platelet-activating factor (PAF) receptor [7]. As a result, they lessen neuronal damage, safeguard neurological function, and enhance patients’ cognitive function. Relevant basic and clinical studies has shown that the diterpene lactones of *G. biloba* have some clinical effects on cognitive impairment [8]. However, no study has yet examined the therapeutic effect of GDLM on cognitive function in patients with acute ischemic stroke (AIS). Based on this, this study examined changes in biochemical measurements, neurological function, and cognitive function in patients with AIS before and after treatment to determine the effectiveness of GDLM injection as an adjuvant therapy to enhance cognitive performance after stroke. If meaningful, the results would provide feasible treatment to ameliorate the cognitive function of AIS patients.

## 2 Materials and methods

### 2.1 Subjects

From July 2019 to December 2020, the AIS patients who were admitted to the Shaanxi Provincial People’s Hospital’s Department of Neurology were continually chosen. Patients ranged in age from 50 to 80. Clinical symptoms, indicators, and imaging data were used to make the diagnosis of AIS. The hospital ethics committee accepted the trial, and each patient signed an informed consent form after providing their informed consent.

The following were the study’s inclusion requirements: (1) The diagnosis of an AIS satisfies the pertinent diagnostic standards outlined in the “Chinese Guidelines for the Diagnosis and Management of Acute Ischemic Stroke 2014” [9]. (2) Hospitalization within 48 h of the stroke’s beginning. (3) No mental impairment prior to the start of AIS. (4) Avoiding using medicines like memantine, piracetam, ginkgo, folic acid, etc., that impair cognitive performance. The following were the exclusion requirements: (1) History of conditions affecting the central nervous system, such as epilepsy, Parkinson’s disease, tumors, brain injuries, encephalitis, strokes, and tumors. (2) Participants with mental or psychological diseases. (3) People suffering from infectious diseases that affect cognitive function, such as hepatitis, HIV, syphilis, and so on. (4) Patients with aphasia, deafness, and blindness who are unable or unwilling to cooperate in completing Montréal Cognitive Assessment (MoCA), Mini-Mental State Examination (MMSE), and other related scoring scales. (5) Patients with severe heart, lung, renal failure, and tumors. (6) Patients who are allergic to GDLM.

General demographic data such as age, gender, education level, past history (hypertension, coronary heart disease, diabetes), and personal history (smoking, drinking) were collected for this study.

### 2.2 Grouping and treatment

The randomized control principle was followed, and all qualified patients were equally divided into two groups: a control group and a treatment group. According to recommendations, all patients should receive conventional care to enhance cerebral blood flow and safeguard nerves, such as statins that lower cholesterol and antiplatelet and anticoagulant medications. In the meantime, manage risk factors such high blood pressure, diabetes, and underlying illnesses. The treatment group received 25 mg of GDLM daily for 14 days straight in addition to the basic care given to the control group.

### 2.3 Observation indicators and clinical efficacy evaluation

(1) Neurological deficits were assessed using the National Institutes of Health Stroke Scale (NIHSS) before treatment and at 14 days and 3 months after treatment. Before and after therapy, patients were evaluated for self-care and daily living skills using the activities of daily living scale (ADL). (2) Before and 14 days after therapy, serum levels of hs-CRP, IL-6, IL-8, fibrinogen (FIB), D-dimer (DD), and platelet (PLT) were assessed in fasting peripheral venous blood. (3) To maximize the sensitivity, cognitive function was assessed using the MMSE and the MoCA. If anyone reached the threshold, it was considered to have post-stroke cognitive impairment (PSCI). Two impartial neurologists who were not connected to the intended intervention evaluated the results prior to therapy, at 3 and 6 months after treatment, and again after these points. According to the correction bias based on years of education, MMSE < 27 points and MoCA < 26 points were considered as PSCI. (4) The latency and amplitude of the P300 evoked potentials were assessed in all patients using a Japanese electromyogram evoked potentiometer before, 3 months after, and 6 months after treatment. (5) Side effects such as gastrointestinal reactions, allergic reactions, liver and kidney function, and bleeding should be monitored after taking the medicine.

### 2.4 Statistical analysis

All of the data collected for this investigation were analyzed using the SPSS 19.0 system software. The study has no bearing on statistics. A *t*-test was used to compare changes in the two groups before and after therapy. Measurement results are expressed as mean standard deviation (*x* ± s). Count statistics are expressed as percentages (%), and *χ* ^2^ or non-parametric tests were used to compare the groups. The cutoff for statistical significance was fixed at 0.05.


**Consent for publication:** Informed consent was obtained from all individual participants included in the study.
**Ethical approval:** All procedures performed in studies involving human participants were in accordance with the 1964 Helsinki declaration and its later amendments or comparable ethical standards. This study is approved by the Ethics Committee of Shaanxi Provincial People’s Hospital. Written informed consent was obtained.

## 3 Results

### 3.1 Demographic and clinical data

The study involved 126 participants in total, and every patient finished the experiment. There were 28 female and 35 males in the therapy group, with a mean age of (67.64 ± 12.32) years. There were 26 female and 37 males in the control group, with a mean age of (68.18 ± 11.53) years. Table 1 displays specifics of the demographic and clinical data. Age, gender, education level, and comorbidities were not statistically different between the two groups, according to the study (*P* > 0.05, Table 1).

**Table 1:** Comparison of demographics and clinical data of patients

Item	Control (*n* = 63)	Treatment (*n* = 63)	*t*/*χ* ^2^-value	*P-*value
Age (years)	67.64 ± 12.32	68.18 ± 11.53	0.965	0.237
Man (*n*%)	36 (57.14)	34 (53.96)	0.239	0.463
Education (years)	9.83 ± 3.92	9.25 ± 4.62	0.873	0.268
Hypertension	41 (65.08)	38 (60.03)	0.453	0.531
Diabetes	34 (53.97)	30 (47.62)	0.954	0.187
CHD	25 (39.69)	28 (44.45)	0.629	0.469
Smoking	28 (44.45)	23 (36.51)	1.289	0.093
Drinking	13 (20.63)	14 (22.23)	0.143	0.711

CHD: coronary heart disease.

### 3.2 Comparison of inflammatory factor levels between two groups pre- and post-treatment

**Figure 1:**
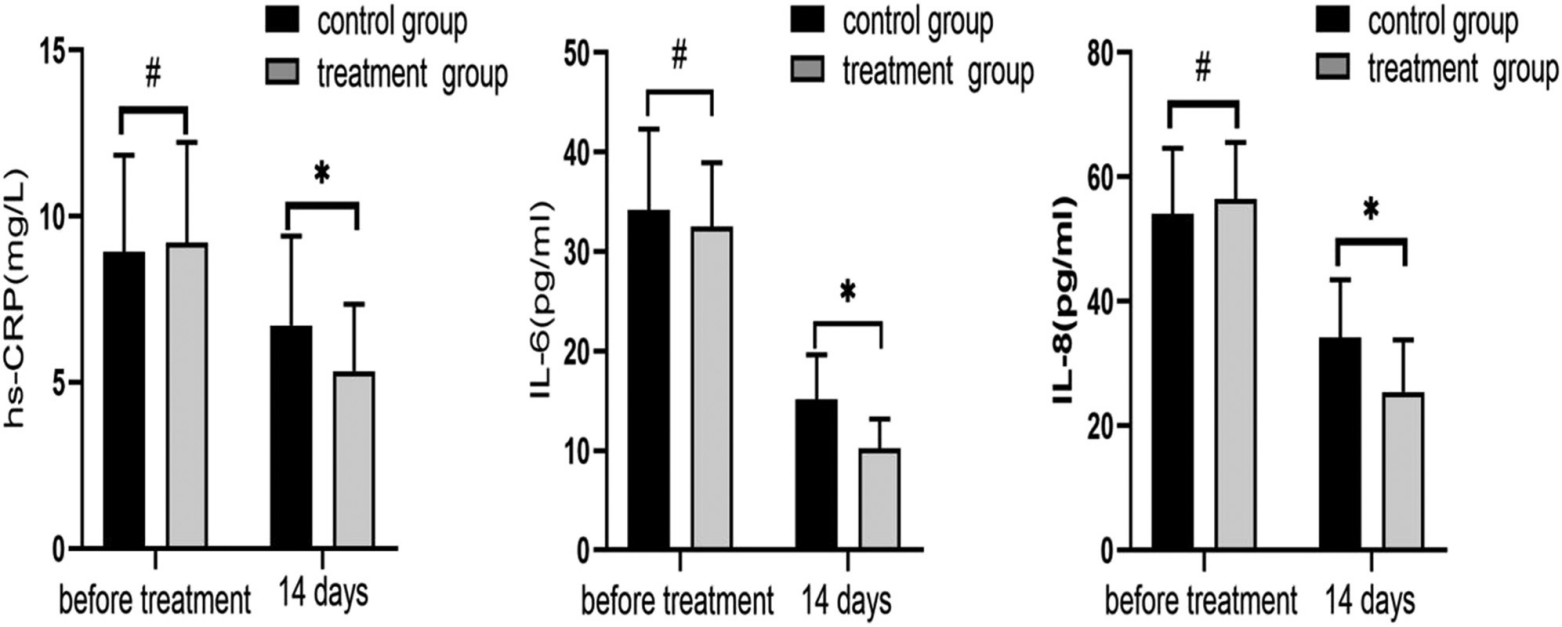
Changes of inflammatory factors hs-CRP, IL-6, and IL-8 before and after 14 days of treatment in both groups. After 14 days of treatment, all indicators decreased, and the indicators in the treatment group were significantly lower than those in the control group (^#^ *P* > 0.05, *P < 0.05, using *t*-test).

According to Figure 1, there was no significant change in serum levels of hs-CRP, IL-6, or IL-8 between the two patient groups prior to treatment (*P* > 0.05). When the aforementioned indicators were retested after 14 days of treatment, the findings revealed that all indicator values fell in both groups, with the treatment group’s indicators being considerably lower than the control group’s (*P* < 0.05).

### 3.3 Comparison of coagulation indexes between two groups pre- and post-treatment

**Figure 2:**
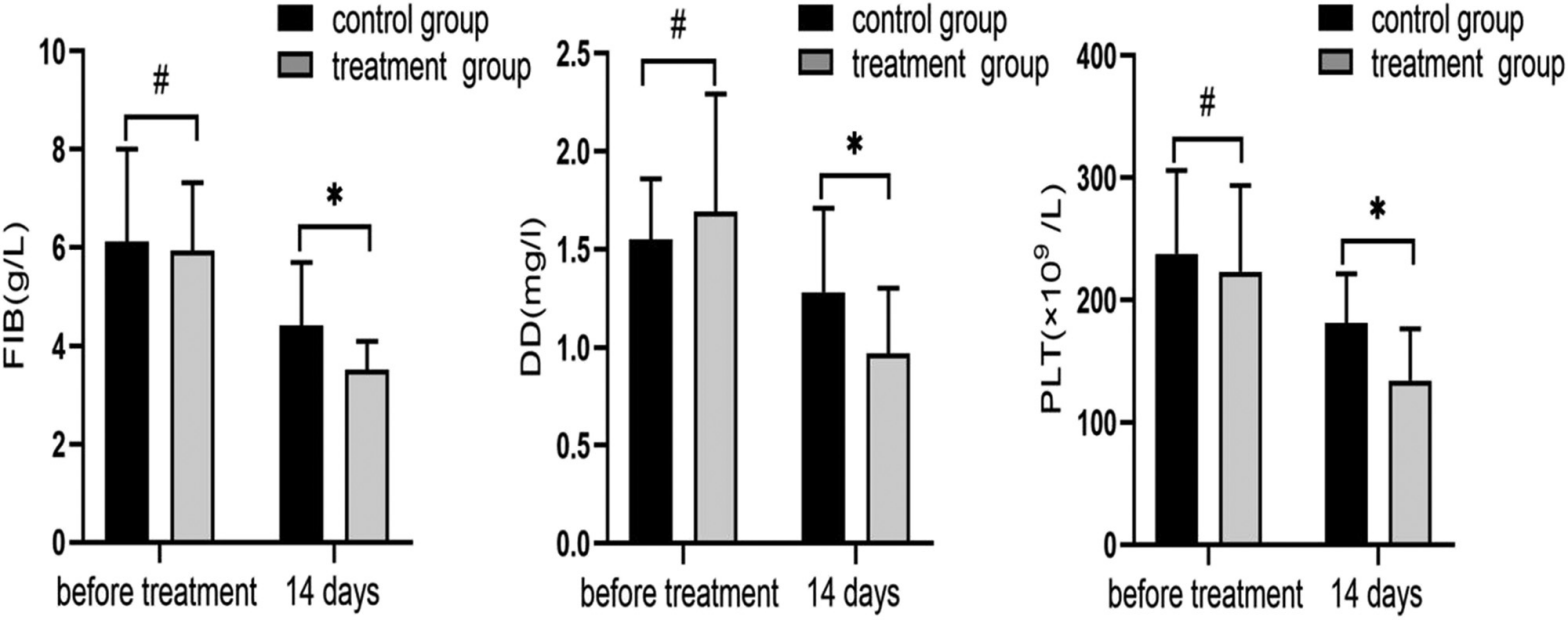
Changes in coagulation indices FIB, DD, and PLT before and 14 days after treatment in both groups. The results of the 14-day post-treatment review showed that all of the above indexes had decreased, and the indexes of the treatment group were significantly lower than those of the control group (^#^ *P* > 0.05, *P < 0.05, using *t*-test).

Before treatment, there was no discernible difference in the two groups’ serum levels of FIB, DD, or PLT (*P* > 0.05). On Day 14 following therapy, the aforementioned parameters were retested. The findings demonstrated that the treatment group’s FIB, DD, and PLT values were lower than those of the control group, and that this difference was statistically significant (*P* < 0.05, Figure 2).

### 3.4 Comparison of neurological deficit and ADL scores between two groups pre- and post-treatment

**Figure 3:**
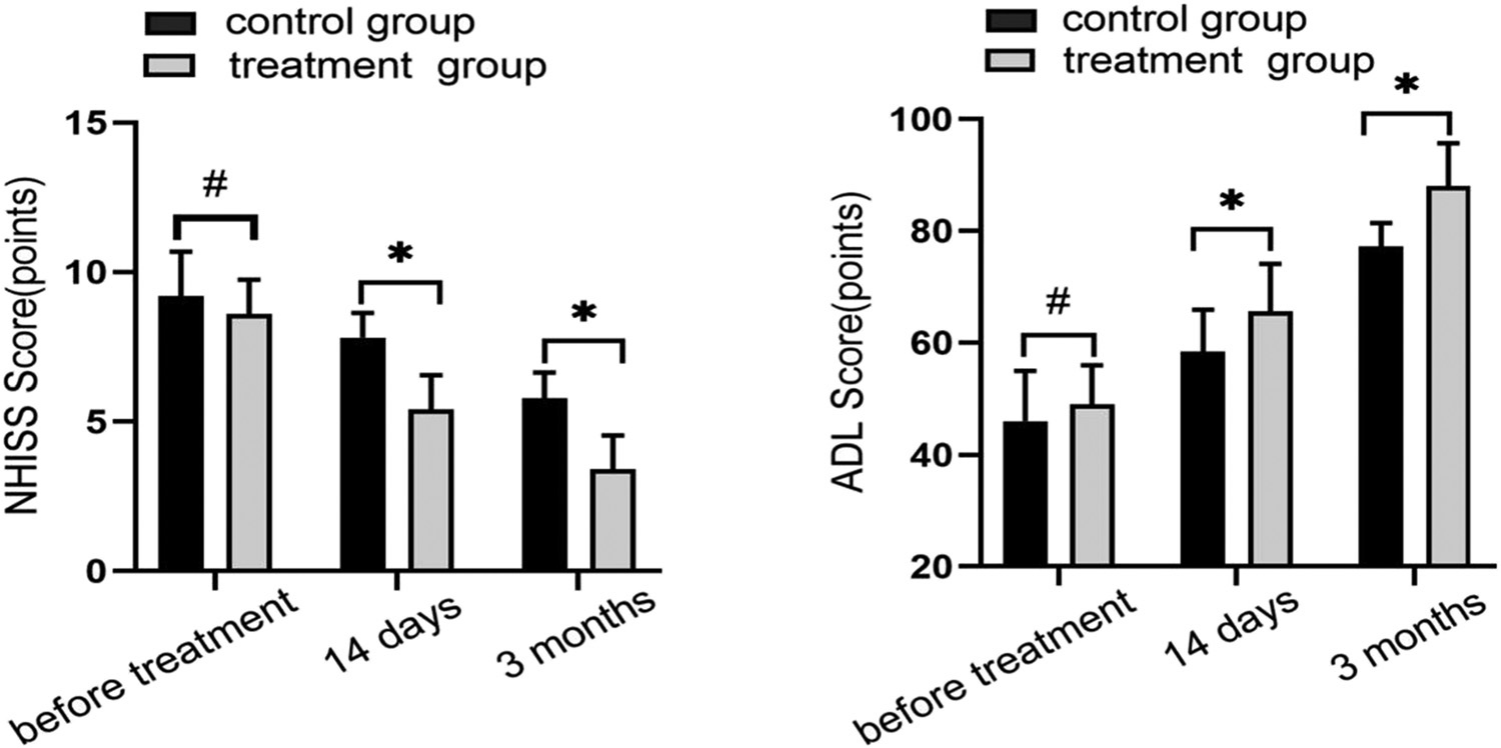
Neurological deficit in functioning (NIHSS) scores and ADL scores were compared between the two groups before treatment, after 14 days of treatment, and after 3 months of treatment. NIHSS scores decreased and were significantly lower than those in the control group at 14 days and 3 months after treatment. ADL scores increased and were significantly higher than those in the control group at 14 days and 3 months after treatment. NIHSS scores increased and were significantly lower than those in the control group at 14 days and 3 months after treatment. NIHSS scores increased and were significantly higher than those in the control group at 14 days and 3 months after treatment (^#^ *P* > 0.05, *P < 0.05, using *t*-test).

The pre-treatment NIHSS scores and ADL scores in both groups did not differ significantly (*P* < 0.05). In both groups, the values of the NIHSS score decreased as time went on, as shown in Figure 3, and were substantially lower in the treatment group than in the control group (*P* < 0.05) after 14 days and 3 months of treatment. At 14 days and 3 months following treatment, the ADL score values were still rising and were higher in the treatment group than in the control group, with the difference being statistically significant (*P* < 0.05).

### 3.5 Comparison of cognitive function assessment between two groups pre- and post-treatment

**Figure 4:**
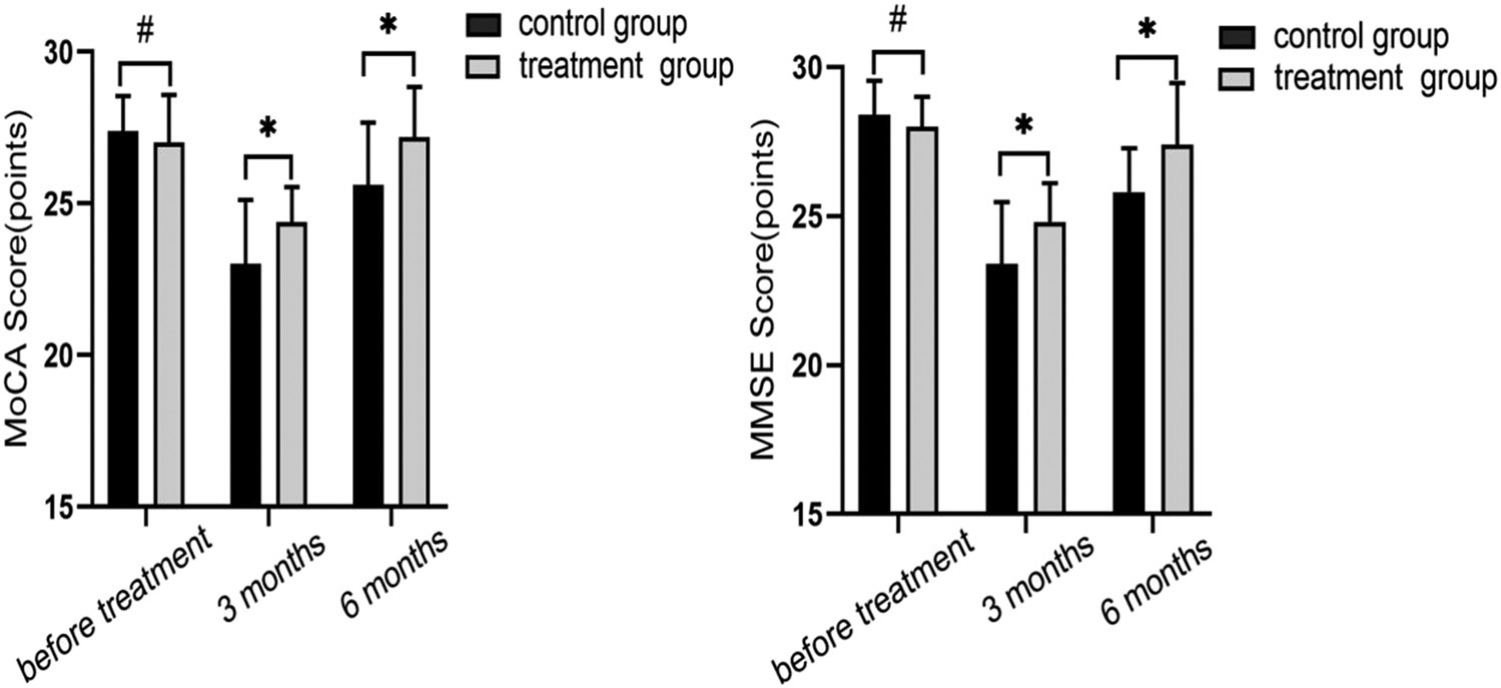
Comparison of cognitive function between the two groups at pre-treatment, 3 months, and 6 months post-treatment. Quantitative comparisons were made using MoCA and MMSE scores. Although there was a decrease in scores at 3 months post-treatment in both groups, scores at 6 months post-treatment were elevated, with the treatment group scoring significantly better than the control group (^#^ *P* > 0.05, *P < 0.05, using *t*-test).

The MoCA and MMSE scores are shown in Figure 4 prior to, 3 months after, and 6 months following therapy, respectively. Prior to therapy, there was no statistically significant difference in the two groups’ MoCA and MMSE scores (*P* > 0.05). Both the MoCA and MMSE scores had dropped in value compared to before the treatment after 3 months. The scores on the MoCA and MMSE were 23.13 ± 1.43 and 24.46 ± 0.98 and 23.47 ± 1.51 and 24.82 ± 1.08 for the two groups, respectively. However, after 6 months of treatment, these scores had improved but were still lower than before treatment. MoCA scores were 25.63 ± 1.25 and 27.22 ± 1.11, and MMSE scores were 25.83 ± 0.93 and 27.22 ± 1.11, respectively. At 3 and 6 months following therapy, respectively, the MMSE and MoCA scores were statistically different between the two groups (*P* > 0.05). It suggests that within 3–6 months after treatment, patients’ cognitive function improved but still did not reach normal levels, with the treatment group showing a better degree of improvement than the control group.

### 3.6 Comparison of event-related potential P300 indicators between two groups pre- and post-treatment

**Figure 5:**
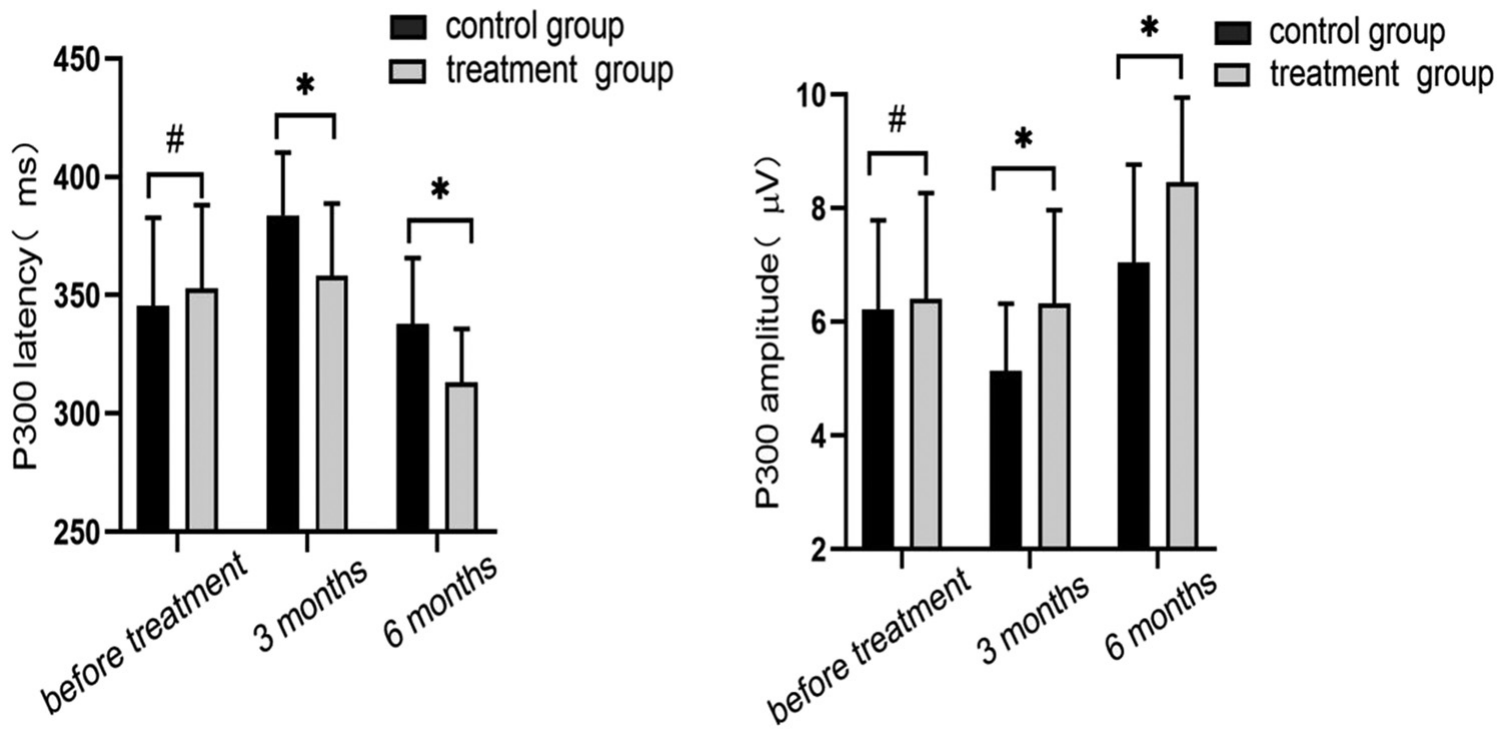
Latency and amplitude of the event-related potential P300 were compared between the two groups before, 3, and 6 months after treatment. After 3 months of treatment, P300 latency was prolonged and amplitude decreased in both groups. After 6 months of treatment, the P300 latency was significantly lower and the amplitude was significantly higher compared with the pre-treatment period. All the treatment groups were better than the control group after treatment (^#^ *P* > 0.05, *P < 0.05, using *t*-test).

There was no statistically significant difference in P300 latency and amplitude between the two groups prior to treatment, as seen in Figure 5. In both groups, the P300 latency increased after 3 months of therapy, but considerably more in the control group than in the treatment group (*P* < 0.05). Similarly, the P300 amplitude decreased in both groups, but again significantly more in the control group than in the treatment group (*P* < 0.05). P300 latency and amplitude were significantly better and less different from pre-treatment in the treatment group than in the control group. After 6 months of treatment, the P300 amplitude of patients in both groups showed an upward trend, and the P300 latency period decreased significantly, and both groups were better than before treatment, and the treatment group was significantly better than the control group (*P* < 0.05).

## 4 Discussion

Stroke the most common cerebrovascular disease, not only has a high mortality rate, but also a high morbidity, recurrence, and disability rate [10]. However, as national public health services and treatment techniques continue to improve, there has been a corresponding increase in stroke survivors and a corresponding increase in the needs of families and patients for stroke prognosis. PSCI is a common complications of AIS. Following a cerebral embolism or cerebral thrombosis, reduced cerebral perfusion, decreased blood oxygen metabolic rate in brain tissue, local inflammatory cytokine and oxygen free radical aggregation, and abnormal neurotransmitter release interact to cause neuronal damage and result in cognitive impairment [11]. It has a significant impact on patients’ neurological recovery and quality of life, and it places a significant financial strain on families and society. In the immediate phase of ischemic stroke, antiplatelet, anticoagulant, and lipid lowering medications are administered to stabilize plaque and alleviate neurological impairments, somewhat lowering the risk of PSCI in stroke patients [12,13]. However, because patients with AIS are more severely ill in the acute phase, most treatment options are focused on improving neurological deficits such as vital signs and motor deficits, while early interventions for cognitive function are often neglected [14,15]. Most patients receive treatment only when vital signs are stable, motor function has recovered and clinical signs of cognitive impairment are evident. Cholinesterase inhibitors, galantamine, memantine, antidepressants, and resveratrol are now frequently used in the clinical management of PSCI and have a good clinical benefit in delaying the progression of the disease [16]. They can give hope to patients and their families; however, the efficacy and safety of these drugs are not satisfactory. The above drugs improve neurological and cognitive function mainly through mechanisms such as enhancing the function of the cholinergic system, reducing neuron excitotoxicity, and decreasing neuroinflammation. But there are no FDA-approved drugs that can control PSCI symptoms or halt the progression of cognitive impairment to dementia in the short term [17]. In view of this, the search for a more effective measure to intervene in cognitive function in the early years after AIS is particularly urgent.

A recent and significant preparation made from *G. biloba*, called GDLM, has been shown to provide a number of clinical benefits, including the removal of blood stasis, the removal of phlegm, the resolution of turbidity, and the reduction of lipids [18]. Studies have shown that it exerts its cerebrovascular protective effects mainly through the following pharmacological pathways [19–21]. GDLM is a PAF receptor antagonist that inhibits platelet aggregation by competitively binding to PAF receptors on abnormally activated platelet membranes and antagonizing the binding of PAF to its receptors. Within platelets, it inhibits [Ca^2+^] release by increasing cAMP levels inhibited by PAF, reducing cellular excitability and protecting neurons. Additionally, GDLM inhibits the phosphoinositide 3-kinase-serine/threonine protein kinase signaling pathway, which prevents intravascular thrombosis and platelet aggregation. After cerebral ischemia, GDLM mitigates damage brought on by inflammation and oxidative stress, lessens production of inflammatory mediators like IL-6, IL-8, and CRP, and avoids oxygen radical-induced cell membrane damage, which lowers the rate of neuronal apoptosis. In addition, GDLM protects cell membranes, reduces the release of excitatory amino acids into the extracellular space after AIS, inhibits cellular metabolism, and attenuates damage caused by hypoxia and glucose deprivation in brain cells [22]. In addition, Li and colleagues [23] found that GDLM could also protect the cerebral vasculature by inhibiting the activation and expression of MMP-9 and reducing the permeability of the blood–brain barrier. Zhou et al. [24] found that GDLM inhibited mitochondrial fission and GSK-3β-dependent increase in mitochondrial membrane permeability, effectively improving I/R injury and alleviating cerebral ischemic injury. The above mechanisms interact with each other, with multiple targets acting together on multiple pathological aspects of AIS to reduce cerebral ischemia and hypoxia damage, protect brain cells, and promote neuronal function repair, thereby reducing learning and memory impairment and improving cognitive function after stroke.

Based on the above and other extensive basic and clinical studies, GDLM has been shown to be neuroprotective and may improve cognitive function in patients with AIS. In this study, it was discovered that patients who received GDLM in addition to conventional treatment showed significantly greater improvement in neurological function than those who received conventional treatment only, with a statistically significant difference between the two groups (*P* < 0.05), by comparing NIHSS and ADL scores of AIS patients before, 14 days, and 3 months after treatment. This shows that GDLM and AIS may work better together to reduce neurovascular injury during acute phase stroke and to enhance AIS prognosis. This might be directly related to how GDLM works to protect the brain. FIB and DD are important human coagulation cofactors that are implicated in thrombosis [25] and are crucial in the etiology of AIS. Our results showed a decrease in FIB and DD levels in both groups compared to pre-treatment, and the changes were more pronounced in the treatment group. It is hypothesized that GDLM may improve cognitive function by lowering the levels of FIB and DD, reducing blood viscosity, improving hypercoagulability and cerebral microcirculation, and promoting the repair of damaged neurons. The prognosis of AIS is significantly impacted by inflammatory variables and oxidative stress, both of which are independent risk factors [26]. In order to lessen brain damage brought on by inflammatory reactions and oxidative stress, GDLM maintains neurological function by preventing the release of inflammatory factors including IL-6 and IL-8 [5]. According to the study, levels of inflammatory mediators such hs-CRP, IL-6, IL-8, and oxygen free radicals were significantly lower in the GDLM group following treatment compared to the control group.

The MMSE assesses seven different cognitive domains, mainly including language, memory, orientation, and numeracy, with a total score of 30; the MoCA scale mainly detects eight domains such as attention, abstract thinking, executive function, and visual structure, with a total score of 30. Using the two together, the coverage is more comprehensive and can truly reflect the level of cognitive function of patients, thus increases the frequency of detection of cognitive impairment and increases the sensitivity and specificity of the assessment [27]. In the current study, 35 of 63 (37.6%) patients in the control group developed coronary artery disease in the sixth month after treatment, consistent with previous reports [28]. In comparison to the control group, the GDLM group had significantly higher MoCA and MMSE scores and a 27.9% reduced PSCI incidence (*P* < 0.05). As a supplement to standard AIS treatment, it is proposed that GDLM plays a part in treating cognitive impairment following AIS. In addition, P300 is an early discovery of an endogenous event-related potential component that can reflect changes in brain neurophysiology and mental activities such as attention, recognition, memory, and judgment during cognition. Its main indicators are latency and amplitude, which indicate the speed and extent to which subjects respond to external input. The degree of cognitive impairment in the brain can therefore be reliably assessed using it in a clinical setting [29]. According to the results of Wang et al. [30], the P300 latency and amplitude in the current investigation increased after treatment in both groups, with the change being higher in the treatment group (*P* < 0.05). Therefore, we hypothesize that GDLM may influence cognitive function after stroke by improving the electroencephalogram response induced by external stimuli.

Although GDLM is a PAF receptor antagonist that acts primarily on abnormally activated platelets, it has no significant effect on normal PLT function or numbers [31]. This study showed statistically significant changes in platelet count before and after treatment, but no cases of serious bleeding were reported and other adverse effects such as gastrointestinal symptoms, liver and kidney impairment and allergic reactions were rare. GDLM is therefore used with a high degree of safety.

There are some shortcomings in this study, the sample size is small, and the collection range is only one hospital in one city, and the age of the sample has some limitations, and the time point of post-treatment testing is not set sufficiently. Therefore, subsequent studies could expand the sample size and age range as much as possible, and set shorter intervals and a larger range of time points, in order to provide more reliable evidence for the clinical treatment of PSCI.

## 5 Conclusions

GDLM combined with regular medication can effectively ameliorate the cognitive function of AIS patients, significantly improve MoCA and MMSE scores in AIS patients by protecting brain cells and promoting neuronal recovery. A reduction in levels of inflammatory cytokines and coagulation markers was also observed. It also has a high safety profile and deserves clinical recommendation. With subsequent multi-center and larger scale, GDLM is expected to be better applied to the treatment of AIS and PSCI.
